# Low iron status and cognitive impairment in northern China community-dwelling older adults: a cross-sectional study

**DOI:** 10.3389/fnut.2025.1672340

**Published:** 2025-11-18

**Authors:** Jimei Xin, Wei Liu, Wensi Li, Qiyue Feng, Gaiping Hao, Lei Wang

**Affiliations:** 1 Department of Epidemiology, School of Public Health, Shanxi Medical University, Jinzhong, Shanxi, China; 2 Third Hospital of Shanxi Medical University, Shanxi Bethune Hospital, Shanxi Academy of Medical Sciences, Tongji Shanxi Hospital, Taiyuan, Shanxi, China; 3 Department of Mathematics and Statistics, Pomona College, Claremont, CA, United States; 4 Beiying Community Health Service Center, Taiyuan, Shanxi, China

**Keywords:** cognitive function, elderly population, risk factors, serum, iron

## Abstract

**Background:**

The association between serum iron concentration and cognitive ageing remains inconsistent, particularly in rural Chinese populations where both iron deficiency and excess may coexist.

**Methods:**

In this cross-sectional analysis of 737 community-dwelling adults aged ≥60 years from rural Taiyuan, China, cognitive impairment was assessed with the Chinese-language Mini-Mental State Examination (MMSE) using education-specific cut-offs. Serum iron was quantified by inductively coupled plasma-mass spectrometry and categorized into tertiles (low, medium, high). Multivariable logistic regression models adjusted for sociodemographic, lifestyle and clinical covariates examined independent and joint associations; restricted cubic splines (RCS) evaluated dose–response patterns.

**Results:**

After comprehensive adjustment, serum iron demonstrated a non-linear relationship with cognitive impairment risk. Relative to the lowest tertile, medium (OR = 0.61, 95% CI: 0.39–0.95) and high (OR = 0.59, 95% CI: 0.37–0.93) serum iron concentrations were associated with reduced odds of cognitive impairment. Spline analysis suggested a U-shaped relationship: risk decreased with rising serum iron up to a nadir and then trended upward; however, the departure from linearity was not statistically significant (*P*-nonlinear = 0.194).

**Conclusion:**

Both low and high serum iron levels are linked to poorer cognitive performance in this rural cohort, supporting the need to maintain iron within an optimal range for healthy cognitive ageing.

## 1 Introduction

According to relevant statistical data, the number of dementia patients globally has reached approximately 50 million, and it is projected that by 2050, this figure will soar sharply to 152 million (1). There are significant differences in the prevalence of cognitive impairment among populations in different countries. The prevalence is 16–20% in the USA and Australia, 5–15% in Germany and Finland (2). In China, the situation of cognitive impairment among the elderly is equally severe. Among adults ≥60 years, 14.5% meet MCI criteria and 4.8% progress to dementia within 7 years (3). Nationwide survey shows that among adults ≥60 years in China, rural mild cognitive impairment prevalence is 1.4-fold higher as well (25.1% vs. 17.9%) (4).

Cognitive impairment is a state in which cognitive functions decline or even are lost due to various factors, with specific manifestations such as memory loss, inattentiveness, difficulty in language expression, and loss of spatial orientation ability (5). From a clinical and neurobiological perspective, cognitive impairment is not the cause but the clinical manifestation of underlying pathological processes that disrupt brain structure and neurotransmitter homeostasis. In Alzheimer’s disease and related tauopathies, hyper-phosphorylation of tau protein detaches it from microtubules, leading to the formation of neurofibrillary tangles, microtubule destabilization, and impaired axonal transport (6). These changes correlate with the severity of cognitive decline rather than being produced by it. The pathological synergy of *β*-amyloid protein (Aβ) can be explained by soluble Aβ oligomers triggering kinases, such as GSK-3β, which further promote tau hyperphosphorylation, while tau pathology promotes Aβ aggregation, forming a feedforward loop, amplifying synaptic dysfunction and neuronal loss (7).

Iron dyshomeostasis is an additional driver. Excess iron catalyzes Fenton chemistry, generating hydroxyl radicals that oxidize lipids, proteins and DNA; this oxidative stress accelerates Aβ plaque formation and tau hyper-phosphorylation (8). Conversely, iron deficiency reduces *α*-secretase activity, indirectly increasing Aβ production (9). Maintaining physiological iron levels is therefore critical for limiting both amyloidogenic and tau-related neurodegeneration. Iron is the most abundant trace metal element in the human body. It plays an irreplaceable role in various aspects of human growth and development, metabolism, immune function, etc., and is a key element for maintaining normal physiological functions of the human body (10, 11). Meanwhile, iron is also involved in the synthesis and metabolism of neurotransmitters such as dopamine and serotonin. These neurotransmitters play significant roles in the information transmission of the nervous system and are closely related to human cognitive functions, emotion regulation, and other aspects (11, 12).

Large-scale epidemiological investigations have found that the incidence of cognitive impairment is higher among people with iron deficiency (13, 14). In patients with iron-deficiency anemia accompanied by cognitive impairment, cognitive function improves after iron supplementation, indicating a correlation between iron deficiency and cognitive problems (15). In view of this, it is of great necessity to conduct in-depth analysis and evaluation of existing research. By thoroughly exploring the correlation between the iron content in the elderly and cognition, and further clarifying the mechanism of iron’s role in cognitive function, it holds practical significance for the early diagnosis and intervention of cognitive impairment in the elderly.

## 2 Materials and methods

### 2.1 Study population

This study was conducted in Taiyuan City, Shanxi Province from May to July 2023, during which rural residents were widely recruited as the research subjects. This study was conducted in Taiyuan City, Shanxi Province from May to July 2023. Participants were recruited during routine community health check-ups organized by Beiying and Huangling Community Health Service Centres (Xiaodian District, Taiyuan, Shanxi). The inclusion criteria are as follows (all of the following conditions need to be met simultaneously): the elderly aged 60 years or above (born before January 1, 1963); being conscious; voluntarily participating, providing informed consent, and signing the informed consent form; having complete baseline data. The exclusion criteria are as follows (those who meet one or more of the following conditions will be excluded): having a history of mental diseases and severe brain diseases; having impaired consciousness, obvious hearing or language function impairments, and being unable to cooperate with the examination; taking medications such as diuretics. After the physical examination, each participant will receive a printed report indicating their serum iron levels and brief dietary guidance for iron deficiency.

Before formal cognitive testing, each participant underwent a brief conversational screen to confirm coherent speech and comprehension. Individuals who appeared drowsy, disoriented, or unable to answer simple questions were excluded as “impaired consciousness.” The orientation subset of MMSE (first 5 items) served as a gatekeeper: anyone scoring <3 was excluded. Village health records were reviewed to pre-identify documented severe mental illness or dementia, and proxy reports were collected when necessary. These steps aimed to exclude delirium and severe cognitive compromise while retaining mild cognitive impairment and normal cognition for analysis. Definitive clinical sub-typing (MCI and dementia) is beyond the scope of this survey and is acknowledged as a limitation.

### 2.2 Cognitive performance tests

The study used the Chinese version of the Mini-Mental State Examination (MMSE) (16) an internationally influential scale widely used in the examination and diagnosis of cognitive impairment, which was employed to assess the mental state of the surveyed subjects (17). This scale is simple and easy to use, making it suitable for large-scale screening. Its content covers the assessment of orientation ability, memory ability, attention and calculation ability, recall ability, and language ability. There are a total of 30 questions in the whole set of the examination. One point can be obtained for each correct answer, and no points will be deducted for wrong answers. The full score is 30 points.

The MMSE was administered entirely orally by three licensed general-practice students. For illiterate participants, the reading item was converted to verbatim repetition and the writing item to spontaneous sentence generation; all other tasks were presented verbally and responses accepted verbally or by gesture, as validated in rural Chinese populations (18).

The diagnosis of cognitive impairment needs to follow strict criteria. Those who meet all the following conditions will be judged as patients with cognitive impairment: (1) The surveyed subjects subjectively feel that their memory has declined, or their family members report that they have obvious memory disorder problems; (2) Through objective testing, it is confirmed that there is an actual situation of memory impairment; (3) The scores of the Mini-mental State Examination (MMSE) are classified and defined according to different educational levels, as follows: For illiterate people, the score is ≤17 points; for those with a primary school education level, the score is ≤20 points; for those with a junior high school education level or above, the score is ≤24 points (16).

### 2.3 Serum iron measurement

Serum specimens were processed, aliquoted, and stored at −80 °C within 2 h of collection. Samples were shipped on dry ice to the Collaborative Laboratory Services, Department of Laboratory Medicine, Shanxi Bethune Hospital (Taiyuan, China). Iron concentration was quantified using a timed-end-point method on an AU-5821 automatic analyzer (Beckman Coulter, USA). In the reaction, iron is released from transferrin by acetate buffer, reduced to the ferrous state by hydroxylamine and thiourea, and immediately complexed with Ferene chromogen. The system monitors the change in absorbance at 560 nm (primary) and 700 nm (secondary) at fixed time intervals; the absorbance change is directly proportional to the iron concentration in the sample.

Each analytical run included a single-level liquid calibrator (80–120 μmol L^−1^ traceable to NIST SRM 1640a) and two-level commercial controls (low/high). Between-day CV was <3%. Samples with a hemolysis index >300 mg L^−1^ or lipaemia >5 mmol L^−1^ were excluded. All pipetting steps were performed in a Class II biological safety cabinet using polypropylene consumables to avoid exogenous metal contamination; background absorbance was verified <0.005 AU before each run. The assay meets ISO 15189:2022 requirements for trace-element analysis.

### 2.4 Covariates

Covariates were chosen by a three-step DAG-guided hierarchical approach (sociodemographic, lifestyle and clinical) (19). Multicollinearity was examined by VIF; all values < 2.2 (mean ≈ 1.4), indicating no concerning multicollinearity. This study included the following covariables: sex; age groups (60–69 years old, 70–79 years old, ≥80 years old); educational level (classified as illiterate, primary school education, junior high school education, senior high school education, undergraduate education); BMI [<18.5 Kg/m (2), 18.5–23.9 Kg/m^2^, 24–27.9 Kg/m^2^, ≥28 Kg/m^2^]; self-assessment of health status of older persons (classified as satisfied, partially, uncertain, slightly, dissatisfied); living alone; physical exercise (never, more than once a day, more than once a week, more than once a month); internet surfing; smoking (never, ever smoked, always smoking); drinking (never, more than once a month, less than once a month, ever drink); diabetes; dyslipidemia; and heart attack.

### 2.5 Statistical analysis

Since the distribution of the Fe element in the human body shows a skewed distribution pattern, the geometric mean (GM), geometric mean-standard deviation (GM-SD), and tertiles (Q1, Q2, Q3) were selected to elaborate in detail the distribution of the Fe element content across various variables. The Mann–Whitney test method was used to explore the influence of the above-mentioned various variables on the distribution of the Fe element level. Logistic regression was applied to analyze the correlation between the serum Fe concentration and the risk of cognitive impairment. In Model 1, no variables were adjusted; in Model 2, variables such as sex, age, educational level, BMI, and self-assessment of health status of older persons were adjusted; based on Model 2, Model 3 further adjusted variables such as living alone, physical exercise, Internet surfing, smoking, drinking, diabetes, dyslipidemia, and heart attack. By means of the restricted cubic spline (RCS), the serum Fe concentration was incorporated into the model as a continuous variable, and then a dose–response curve was plotted to visually present the dose–response relationship between the serum Fe concentration and relevant factors. Serum iron concentrations were log₁₀-transformed to normalize right-skewed distribution and meet linearity assumptions of restricted cubic spline regression. Statistical analysis software such as SPSS 24.0 and R 4.4.1 were used to conduct a comprehensive statistical analysis of the collected data. *P* ≤ 0.05 was considered to be statistically significant.

## 3 Results

### 3.1 The distribution of serum iron levels of the study population

Table 1 lists the basic information of the research subjects. In this study, a total of 800 research subjects met the conditions of this study. After excluding the participants who did not provide the data of serum iron concentration, 737 adults aged 60 and above were finally included in this study. The geometric mean (GM) of the serum Fe concentration in the total population was 17.65 μmol/L, indicating that the overall serum Fe concentration was around this level; the geometric mean-standard deviation (GM-SD) was 1.69 μmol/L, which reflected the degree of dispersion of the data around the geometric mean. The study found that there were significant differences in the serum Fe concentration among different sexes, smoking statuses, drinking statuses, and cognitive impairment statuses (*p* < 0.05).

**Table 1:** The distribution of serum Fe concentration (μmol/L) in the general demographic characteristics of the elderly.

Characteristic	*N*	GM	GM-SD	Fe			*P*
				Q_1_	Q_2_	Q_3_	
*N*	737	17.65	1.69	11.61	18.59	25.52	
Sex							< **0.001**
Male	304	18.98	1.63	11.53	18.71	26.30	
Female	433	16.78	1.72	11.66	18.52	24.60	
Age(y)							0.059
60–69	359	18.46	1.56	12.41	18.72	25.67	
70–79	324	16.98	1.79	11.32	18.43	25.22	
≥80	54	16.51	1.93	8.60	18.75	26.05	
Educational level							0.100
Illiteracy	117	16.74	1.47	11.70	18.40	25.23	
Primary school	338	17.57	1.77	11.11	18.52	25.36	
Junior high	215	18.23	1.77	11.86	18.72	25.99	
Senior high and above	67	17.82	1.34	13.05	18.88	25.08	
BMI(Kg/m^2^)							0.228
<18.5	18	16.88	1.58	11.16	18.85	24.97	
18.5–23.9	234	17.36	1.75	11.07	18.57	25.60	
24.0–27.9	339	17.23	1.78	11.27	18.61	25.59	
≥28.0	146	19.29	1.33	13.82	18.50	25.37	
Self-assessment of health status of older persons							0.261
Satisfied	420	17.85	1.66	11.42	18.72	25.56	
Partially	255	17.90	1.60	12.39	18.44	25.55	
Dissatisfied	62	15.46	2.17	10.30	18.32	25.02	
Living alone							0.211
No	577	17.95	1.62	11.93	18.63	25.68	
Yes	160	16.62	1.91	10.70	18.38	24.99	
Physical exercise							0.562
Never	115	17.55	1.83	10.94	18.68	25.82	
≥1 daily	426	17.53	1.77	11.41	18.62	25.80	
≥1 weekly	129	18.43	1.35	12.76	18.58	24.36	
≥1 monthly	67	17.17	1.47	12.17	18.15	25.82	
Internet surfing							0.276
No	267	17.23	1.73	11.21	18.46	25.80	
Yes	470	17.90	1.66	11.87	18.66	25.38	
Smoking							**0.004**
Never	505	16.99	1.79	11.27	18.59	25.42	
Ever smoked	77	19.12	1.40	12.38	18.67	25.49	
Always smoking	155	19.20	1.44	12.70	18.49	25.75	
Drinking							**0.007**
Never	553	17.25	1.71	11.72	18.57	25.07	
More than once a month	80	19.90	1.50	11.66	18.71	27.32	
Less than once a month	45	19.01	1.45	12.21	18.29	26.03	
Ever drink	59	17.60	1.83	9.92	18.75	25.50	
Diabetes							0.433
No	593	17.82	1.63	11.75	18.62	25.53	
Yes	144	16.99	1.92	11.11	18.43	25.52	
Dyslipidemia							0.163
No	497	18.17	1.55	12.33	18.53	25.75	
Yes	240	16.62	1.95	10.36	18.70	25.02	
Heart attack							0.196
No	585	17.86	1.67	11.79	18.53	25.55	
Yes	152	16.87	1.77	10.99	18.77	25.40	
Cognitive impairment							**0.013**
No	559	18.03	1.68	11.86	18.59	25.57	
Yes	178	16.50	1.72	11.03	18.58	25.34	

Bold values indicate statistical significance (*p* < 0.05).

### 3.2 Univariate logistic regression analysis of serum iron and cognitive impairment

The research data shows that the serum Fe concentration can affect the risk of cognitive impairment in the elderly. Taking the elderly with low Fe concentration as the reference, there is a statistical association between the moderate Fe concentration and the risk of cognitive impairment in the elderly (*p* < 0.05). Specifically, the risk of cognitive impairment in the elderly with moderate Fe concentration (OR = 0.664, 95% CI: 0.441–0.999) is lower than that in the elderly with low Fe concentration; that is, the risk of suffering from cognitive impairment is lower. For details, please refer to Table 2.

**Table 2:** Univariate logistic regression analysis of the relationship between serum Fe concentration and cognitive impairment.

Characteristic	OR_1_(95%CI)	*P*
Fe level		
Q_1_	Reference	
Q_2_	0.664(0.441–0.999)	**0.049**
Q_3_	0.667(0.443–1.005)	0.053

OR: Odds ratio; OR_1_ refers to the risk of cognitive impairment calculated when no variables are adjusted in Model 1; Q_1_ represents the low Fe concentration level; Q_2_ represents the moderate Fe concentration level; Q_3_ represents the high Fe concentration level; The low concentration group Q1 is used as the reference group.

Bold values indicate statistical significance (*p* < 0.05).

### 3.3 Multivariate logistic regression analysis of serum iron and cognitive impairment

In Model 2, after adjusting for factors such as sex, age, educational level, BMI, and self-assessment of health status of older persons, the study found that compared with the elderly with low Fe concentration, there was a significant statistical association between the elderly with moderate Fe concentration and the risk of cognitive impairment (OR2 = 0.649, 95% CI: 0.432–0.995). Similarly, there was also a statistical association between the elderly with high Fe concentration and cognitive impairment (OR2 = 0.619, 95% CI: 0.401–0.957). This clearly indicates that the risk of cognitive impairment in the elderly with moderate and high Fe concentrations is lower than that in the elderly with low Fe concentrations.

In Model 3, on the basis of Model 2, variables such as living alone, physical exercise, Internet surfing, smoking, drinking, diabetes, dyslipidemia, and heart attack were further adjusted. The results showed that compared with the low Fe concentration, there was still a statistical association between the moderate Fe concentration and cognitive impairment (OR3 = 0.607, 95% CI: 0.388–0.949); there was also a significant statistical association between the high Fe concentration and cognitive impairment (OR3 = 0.591, 95% CI: 0.376–0.928). This further proves that the influence of serum Fe concentration on the risk of cognitive impairment is still prominent, and it always shows that the risk of cognitive impairment in the elderly with moderate and high Fe concentrations is lower than that in the elderly with low Fe concentration.

In addition, the study also revealed the relationship between other factors and the risk of cognitive impairment in the elderly. Among them, elderly men, those of advanced age with a low educational level who do not like surfing the Internet, have never smoked, and do not suffer from diabetes and dyslipidemia, have a relatively higher risk of cognitive impairment, and these differences are statistically significant (*p* < 0.05). For details, please refer to Table 3.

**Table 3:** Multivariate logistic regression analysis of the relationship between serum Fe concentration and cognitive impairment.

Characteristic	OR_2_(95%CI)	*P*	OR_3_(95%CI)	*P*
Fe level				
Q_1_	Reference		Reference	
Q_2_	0.649(0.423–0.995)	**0.047**	0.607(0.388–0.949)	**0.028**
Q_3_	0.619(0.401–0.957)	**0.031**	0.591(0.376–0.928)	**0.022**
Sex				
Male	Reference		Reference	
Female	0.686(0.474–0.994)	**0.046**	0.885(0.495–1.583)	0.680
Age(y)				
60–69	Reference		Reference	
70–79	1.199(0.823–1.747)	0.345	1.044(0.694–1.568)	0.838
≥80	2.321(1.209–4.456)	**0.011**	1.907(0.901–4.037)	0.092
Educational level				
Illiteracy	Reference		Reference	
Primary school	0.430(0.270–0.685)	< **0.001**	0.479(0.292–0.787)	**0.004**
Junior high	0.355(0.210–0.600)	< **0.001**	0.519(0.285–0.945)	**0.032**
Senior high and above	0.182(0.078–0.427)	< **0.001**	0.256(0.100–0.657)	**0.005**
BMI(Kg/m^2^)				
<18.5	Reference		Reference	
18.5–23.9	0.909(0.255–3.243)	0.884	0.778(0.214–2.828)	0.702
24.0–27.9	1.277(0.363–4.484)	0.703	1.102(0.306–3.969)	0.882
≥28.0	1.740(0.480–6.307)	0.399	1.501(0.403–5.592)	0.545
Self-assessment of health status of older persons				
Satisfied	Reference		Reference	
Partially	0.782(0.529–1.155)	0.216	0.779(0.517–1.175)	0.234
Dissatisfied	0.798(0.411–1.552)	0.506	0.788(0.394–1.576)	0.501
Living alone				
No			Reference	
Yes	——		1.020(0.512–2.032)	0.954
Physical exercise				
Never			Reference	
≥1 daily	——		0.718(0.431–1.197)	0.204
≥1 weekly	——		0.933(0.502–1.734)	0.826
≥1 monthly	——		0.699(0.338–1.444)	0.333
Internet surfing				
No			Reference	
Yes	——		0.557(0.371–0.838)	**0.005**
Smoking				
Never			Reference	
Ever smoked	——		0.610(0.243–1.534)	0.294
Always smoking	——		1.487(0.797–2.775)	0.212
Drinking				
Never			Reference	
More than once a month	——		1.170(0.587–2.330)	0.655
Less than once a month	——		1.415(0.607–3.298)	0.421
Ever drink	——		2.540(1.064–6.067)	**0.036**
Diabetes				
No			Reference	
Yes	——		0.553(0.333–0.916)	**0.022**
Dyslipidemia				
No			Reference	
Yes	——		1.752(1.175–2.613)	**0.006**
Heart attack				
No			Reference	
Yes	——		0.885(0.548–1.429)	0.618

OR: Odds ratio; OR_1_: It is the risk of cognitive impairment calculated in Model 1 without adjusting any variables. OR_2_: It refers to the risk of cognitive impairment calculated in Model 2 after adjusting variables such as sex, age, educational level, BMI, and self-assessment of health status of older persons. OR_3_: On the basis of Model 2, it is the risk of cognitive impairment calculated after further incorporating and adjusting variables such as living alone, physical exercise, Internet surfing, smoking, drinking, diabetes, dyslipidemia, and heart attack. Q_1_ represents the low Fe concentration level; Q_2_ represents the moderate Fe concentration level; Q_3_ represents the high Fe concentration level. All use Q_1_ (the low Fe concentration group) as the reference group.

Bold values indicate statistical significance (*p* < 0.05).

### 3.4 Dose–response relationship

**Figure 1:**
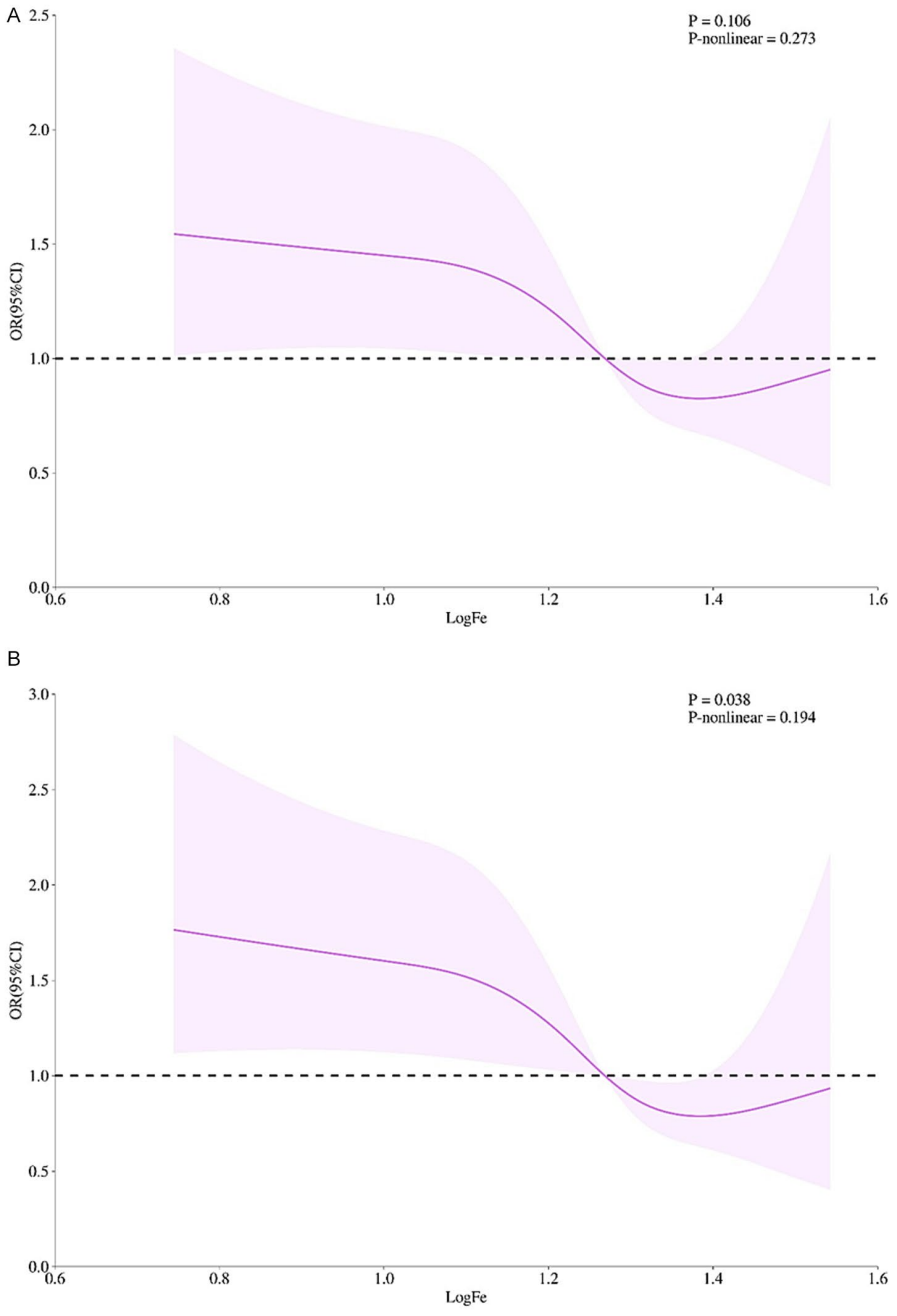
The dose–response relationship between serum Fe level and cognitive impairment in the elderly. **(A)** Unadjusted model. **(B)** Adjusted for sociodemographics (sex, age, educational level), lifestyle (BMI, self-rated health, living alone, exercise frequency, internet use, smoking, alcohol consumption), and comorbidities (diabetes, dyslipidemia, myocardial infarction). The x-axis represents log-transformed serum iron (logFe); the y-axis represents odds ratios referenced to 10 μmol/L. Solid lines denote fitted relationships; shaded areas denote 95% confidence intervals. Panel A shows a U-shaped association with nadir at 16–20 μmol/L (logFe = 1.2–1.3) but a non-significant non-linear term. In panel B, the U-shape was attenuated; the overall trend remained significant (*p* = 0.038) but non-linearity persisted (*p*-nonlinear = 0.194). *n* = [Sample Size]. Splines used 3 knots at the 10th, 50th, and 90th percentiles.

Serum iron showed an approximately “U”-shaped relationship with cognitive impairment risk, which was attenuated after multivariable adjustment. Without adjustment for other variables, risk declined progressively with increasing iron up to 16–20 μmol/L (logFe = 1.2–1.3); beyond this threshold the curve re-ascended, but the departure from linearity did not reach statistical significance (see Figure 1A for details). After further incorporating and adjusting variables, such as sex, age, educational level, BMI, self-assessment of health status of older persons, whether living alone, frequency of physical exercise, Internet surfing, smoking, drinking, as well as diabetes, dyslipidemia, and heart attack, the same nadir is retained in Figure 1B, with the overall trend remaining significant (*p* = 0.038) yet the non-linear component non-significant (*P*-non-linear = 0.194).

## 4 Discussion

Unlike previous studies conducted in urban or Western populations, the study focuses on rural elderly in Shanxi, China, where regional dietary patterns—such as frequent consumption of red dates—may influence iron status. Residents not only often eat red dates directly as daily snacks but also are accustomed to adding red date powder when making traditional foods such as noodles, leading to a relatively high intake of red dates and thus having a significant impact on the serum iron level (20). This population is underrepresented in global cognitive aging research. In contrast, the dietary structure of residents in Shanghai is characterized by a relatively high proportion of animal-derived iron intake, and there may be special combinations in the dietary collocation that promote iron absorption (21). Changes in serum iron levels due to regional dietary differences provide a valuable basis for comparing regional diets, facilitating in-depth exploration of the relationship between iron and human health.

In previous studies, many scholars have carried out extensive and in-depth discussions on the relationship between serum iron and cognitive function. However, the research conclusions vary widely. Some studies have pointed out that iron overload may damage the nervous system through complex mechanisms such as oxidative stress, thereby increasing the risk of cognitive impairment (22, 23). However, some studies did not observe significant associations in specific subgroups (24–26), or due to interference from various factors such as genetics (24), gender and age (24), and other trace elements (25, 27), the research results are complex and difficult to draw clear conclusions. We acknowledge these null findings and provide two explanations: detection method variations and regional dietary habits. Detection method differences influence iron measurement results differently; some advanced detection technologies can precisely measure trace serum iron ions, while the detection method adopted in this study may be lacking in the precision of the lower detection limit or anti-interference ability, causing measurement deviations (28).

This study used logistic regression to examine the relationship between serum iron concentration and cognitive impairment in older adults. After adjustment for a comprehensive set of potential confounders—including age, sex, education, BMI, lifestyle factors and comorbidities—individuals with moderate or high serum iron levels had a significantly lower risk of cognitive impairment than those with low levels. These findings suggest a protective effect of higher iron concentrations within the observed range. Dose–response analysis further revealed an approximately U-shaped association, with the nadir of risk occurring at 16–20 μmol/L. Although the non-linear component lost statistical significance after full adjustment, the overall trend remained significant (*p* = 0.038), supporting a concentration-dependent relationship. This pattern aligns with previous work: a longitudinal study reported that low serum iron was linked to increased risk of post-stroke cognitive impairment (PSCI) (OR = 2.498), whereas high serum iron was associated with reduced risk (OR = 0.368), implying a U-shaped or threshold effect (24). Given the cross-sectional design, we cannot rule out a reverse causal relationship (i.e., subclinical cognitive decline altering dietary iron intake or metabolism). Longitudinal and mechanistic studies are needed in the later stage to determine the optimal iron range for cognitive protection in the elderly.

Regarding the phenomenon that a high iron concentration does not increase the risk of cognitive impairment, it may be due to the exquisite compensatory mechanisms within the body. The absence of excess risk at high-normal iron is compatible with hepcidin-mediated ferroportin internalization and IRP/IRE translational repression, which jointly restrain the labile iron pool, together with ferritin-induction and co-ordinated antioxidant enzymes that blunt Fenton chemistry–derived oxidative stress (29–31). As these mechanisms were neither measured nor validated in the present study, generalizability to other populations is limited, and extension to urban or non-Asian groups is not justified. Beyond global cognition, social-cognitive abilities—emotion recognition and theory of mind—decline with age and may be modulated by iron-dependent dopaminergic signaling (32–34). Therefore, this phenomenon warrants integrated, multi-level studies combining molecular, neuroimaging and epidemiological approaches to clarify iron–cognition links (35). The absence of ferritin, transferrin saturation and hemoglobin data precludes distinction between iron-deficiency anemia, functional iron deficiency and anemia of chronic disease. Future studies incorporating these indicators are needed to clarify the underlying iron-metabolic phenotype driving the observed cognitive associations.

While this study focused on serum iron, we recognize that other trace elements (e.g., zinc, copper, selenium) also influence cognitive function. Future studies should assess trace element profiles to explore synergistic or antagonistic effects on cognition.

## Data Availability

The datasets presented in this article are not readily available due to the confidentiality and privacy of the participants. Requests to access the datasets should be directed to lw10286@163.com.
